# Comparative Study of a Series of ^99m^Tc(CO)_3_ Mannosylated Dextran Derivatives for Sentinel Lymph Node Detection

**DOI:** 10.3390/molecules26164797

**Published:** 2021-08-07

**Authors:** Afroditi Papasavva, Antonio Shegani, Christos Kiritsis, Ioanna Roupa, Myrto Ischyropoulou, Konstantina Makrypidi, Irineos Pilatis, George Loudos, Maria Pelecanou, Minas Papadopoulos, Ioannis Pirmettis

**Affiliations:** 1 Institute of Nuclear and Radiological Sciences and Technology, Energy & Safety, NCSR “Demokritos”, 15310 Athens, Greece; papasavvaphro@gmail.com (A.P.); antonio.shegani@kcl.ac.uk (A.S.); kiritsis.chr@gmail.com (C.K.); iroupa@rrp.demokritos.gr (I.R.); myrto7@gmail.com (M.I.); kmakripidi@rrp.demokritos.gr (K.M.); mspap@rrp.demokritos.gr (M.P.); 2 BIOEMTECH Laboratories, Lefkippos Attica Technology Park—NCSR “Demokritos”, 15310 Athens, Greece; ipilatis@bioemtech.com (I.P.); george@bioemtech.com (G.L.); 3 Institute of Biosciences & Applications, NCSR “Demokritos”, 15310 Athens, Greece; pelmar@bio.demokritos.gr

**Keywords:** sentinel lymph node, dextran, mannose, cysteine, technetium-99m, SPECT imaging

## Abstract

Sentinel lymph node detection (SLND) is rapidly entering common practice in the management of patients with tumors. The introduction of mannose molecules to ^99m^Tc-labeled dextrans, so far, showed that the sentinel node could trap these agents due to their recognition by the mannose receptors of lymph node macrophages. The current study aimed to synthesize, characterize, and biologically evaluate a series of mannosylated dextran derivatives labeled with ^99m^Tc for potential use in SLND. The compounds were designed to have a dextran with a molecular weight of 10–500 kDa as a backbone, S-derivatized cysteines, efficient SNO chelators, and mannose moieties for binding to mannose receptors. They were successfully synthesized, thoroughly characterized using NMR techniques, and labeled with the *fac*-[^99m^Tc(CO)_3_]^+^ synthon. Labeling with high yields and radiochemical purities was achieved with all derivatives. In vivo biodistribution and imaging studies demonstrated high uptake in the first lymph node and low uptakes in the following node and confirmed the ability to visualize the SLN. Among the compounds studied, ^99m^Tc-D75CM demonstrated the most attractive biological features, and in combination with the high radiochemical yield and stability of the compound, its further evaluation as a new radiopharmaceutical for sentinel lymph node detection was justified.

## 1. Introduction

Over the last decade, research on developing new radiopharmaceuticals has focused on the selective binding of a radiolabeled biomolecule to a receptor. Specifically, the biomolecule acts as a vector that transports the radionuclide to the tissue overexpressing the receptor to enable imaging and diagnosis with a SPECT or PET camera or with radiotherapy [1]. Among the SPECT isotopes currently in use, technetium-99m (^99m^Tc) remains the radionuclide of choice, having excellent physicochemical properties, easy access through generators, and low cost compared with radionuclides, the production of which requires a cyclotron (^18^F, ^123^I, ^111^In, ^11^C, ^13^N, ^15^O) [2].

The development of diagnostic radiopharmaceuticals for the precise localization of the sentinel lymph node (SLN), the hypothetical first lymph node to receive lymph and metastatic cells from the primary site of the tumor, is actively explored, aiming at identifying affected tissues that require surgical removal. The metastatic pathway of some cancers usually follows a course that begins from the nearby lymph nodes. Therefore, if the first draining lymph node, the SLN, is negative in tumor metastasis, the presence of cancerous cells in all other lymph nodes is highly improbable. Thus, sentinel lymph node biopsy (SLNB) gradually replaces extensive lymph node removal in patients with cancer, offering more accurate diagnosis and reducing unnecessary lymph node dissection (lymphadenectomy) [3,4,5,6,7].

Sentinel lymph node detection (SLND) in nuclear medicine is performed by injecting small, radiolabeled particles in the tumor area. The particles migrate from the injection site into the lymphatic system mainly by passive diffusion and are cleared from the lymph as a foreign matter through active saturable phagocytosis. Particle size influences the rate of colloid drainage from the injection site to the dermal lymphatic capillaries as well as phagocytosis by lymph node macrophages. Particles larger than 100 nm are trapped in the interstitial space, resulting in masking of the SLNs, whereas smaller particles clear faster from the injection site but may leak to the bloodstream (<5 nm) and distant lymph nodes. A number of ^99m^Tc colloidal radiopharmaceuticals are clinically used (filtered ^99m^Tc-sulfur colloid, ^99m^Tc-antimony, and various ^99m^Tc-labeled albumin preparations); however, they are associated with certain drawbacks, such as high injection site retention, migration to subsequent higher echelon lymph nodes, as well as adverse effects associated with the employment of materials of biological origin, such as gelatine or human albumin [8,9].

An alternative direction for developing radiopharmaceuticals for SLND targets the mannose receptors on the surface of the macrophage cells present in the lymph node [10,11,12,13,14]. A series of mannosylated dextrans have been explored in that direction, taking advantage of the nanoparticle nature of dextrans and their suitability for biological applications, properties such as being water soluble, biodegradable, non-toxic, and available in various molecular weights [15,16,17,18,19,20]. This direction resulted in the development of ^99m^Tc-tilmanocept, the first authorized radiopharmaceutical in Europe and USA to selectively localize the SLN by binding to the mannose receptor (CD206). The radiotracer is a synthetic macromolecule composed of a 10 kDa dextran backbone, several DTPA (diethylenetriaminepentaacetic acid) units serving as attachment sites for ^99m^Tc chelation, and mannose for receptor binding. However, DTPA is not considered an ideal chelator for ^99m^Tc, and the structure of its ^99m^Tc-complexes is not well defined. In addition, the selectivity of ^99m^Tc-tilmanocept for the SLN is not optimal, as its transport in secondary nodes is notable, while a high percentage of the agent remains at the injection site. Thus, it is of particular interest to prepare well-characterized complexes showing fast injection site clearance; fast, high, and persistent uptake by the SLN; and low leakage to higher echelon nodes, together with a favorable safety profile [15,18,19,20,21,22].

Intending to develop SLND imaging agents with improved characteristics, our laboratory has previously reported the development of a novel ^99m^Tc-radiolabeled mannosylated dextran derivative, ^99m^Tc(CO)_3_-DCM20, with a 10 kDa dextran backbone bearing S-derivatized cysteines as efficient SNO chelators for the *fac*-[^99m^Tc(CO)_3_]^+^ core and with mannose moieties for binding to mannose receptors of the lymph node. The ^99m^Tc(CO)_3_-DCM20 derivative proved to be stable and of high radiochemical purity and specific activity, while its biological evaluation showed fast injection site clearance and high and persistent uptake in the SLN [23,24]. Expanding this promising project, we present herein the synthesis and biological evaluation of a series of DCM derivatives employing dextrans of different MW spanning 10–500 kDa in an attempt to develop a ^99m^Tc-DCM product with improved biological characteristics as an SLN imaging agent.

## 2. Results

### 2.1. Synthesis and Characterization of the Mannosylated Dextran Derivatives

**Scheme 1:**
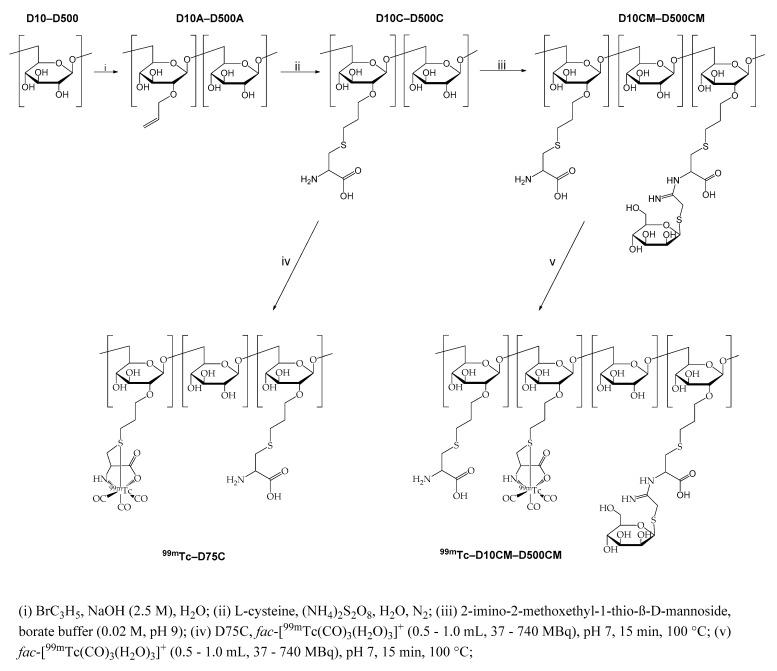
Synthesis of D10CM–D500CM and preparation of ^99m^Tc-complexes ^99m^Tc-D75C and ^99m^Tc-D10CM–^99m^Tc-D500CM.

The new dextran derivatives D10CM–D500CM were synthesized and characterized by NMR following the procedures described in detail for DCM20 [23]. Specifically, the reaction of allyl bromide with dextrans D10–D500 (MW 10–500 kDa) yielded the intermediate allyl dextrans D10A–D500A, Scheme 1. Comparing the intensity of allyl peaks in the NMR spectra with those of the anomeric protons of dextran indicated that 21–35% of the dextran glucose units were allylated. Addition of cysteine to allyl dextran resulted in the quantitative formation of the dextran–cysteine derivatives (D10C–D500C). Subsequently, D10C–D500C reacted with the bifunctional reagent 2-imino-2-methoxethyl-1-thio-β-D-mannopyranoside to yield the mannosylated dextran derivatives (D10CM–D500CM). Comparison of the intensity of the anomeric protons of mannose to that of the anomeric protons of dextran showed that 53–82% of the cysteines were mannosylated. The number of cysteines and mannose units present in each DCM derivative based on NMR data are presented in Table 1. The ^1^H NMR spectra of D75A, D75C, and D75CM are given in Supplementary Materials (Figure S1) as representative of the spectra obtained with the different MW dextrans, together with a stack plot of the ^1^HNMR spectra of the rest of the mannosylated dextrans (Figure S2).

### 2.2. Radiolabeling

Radiolabeling was effected through the employment of the ^99m^Tc(CO)_3_ tricarbonyl core. In-house production of the *fac*-[^99m^Tc(CO)_3_(H_2_O)_3_]^+^ precursor was performed by direct addition of ^99m^TcO_4_^−^ in a closed vial in the presence of CO gas and NaBH_4_ as a reducing agent and by heating for 30 min at 95 °C, followed by adjustment of the pH to 7. The quality control of the radiolabeling by HPLC showed more than 97% formation of the precursor.

**Figure 1:**
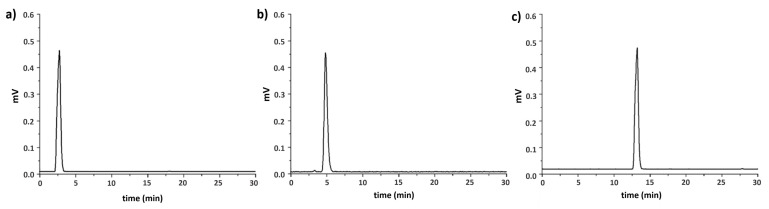
RP-HPLC chromatograms (*γ*-detection) of (**a**) ^99m^TcO_4_^−^, (**b**) *fac*-[^99m^Tc(CO)_3_(H_2_O)_3_]^+^, and (**c**) ^99m^Tc−D40CM.

The mannosylated compounds, as well as the non-mannosylated D75C derivative, were labeled with *fac*-[^99m^Tc(CO)_3_(H_2_O)_3_]^+^ precursor at low ligand concentration (1.2 × 10^–7^ to 4.4 × 10^–6^ M) and heating for 15 min at 100 °C. In each case, HPLC analysis of the radiolabeling reaction mixture showed the presence of a single peak for each complex (^99m^Tc-D10CM, ^99m^Tc-D500CM), eluting at 12 to 13 min, which was distanced from the *fac*-[^99m^Tc(CO)_3_(H_2_O)_3_]^+^ precursor eluting out at 5.1 min and the ^99m^TcO_4_^−^ eluting at 2.6 min (Figure 1).

**Figure 2:**
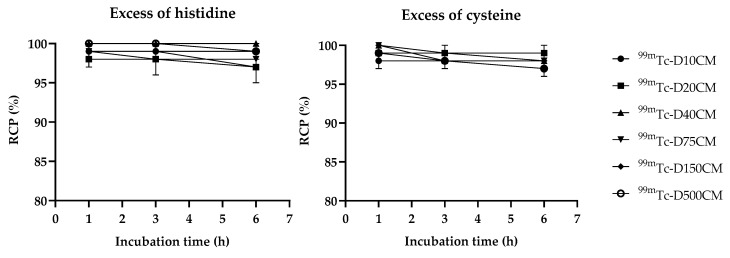
In vitro stability of ^99m^Tc−D10CM–^99m^Tc−D500CM in the presence of excess histidine and cysteine (10^−2^ M) at 37 °C for 1, 3, and 6 h.

Stability studies showed that the ^99m^Tc-complexes remained stable in their reaction mixture for up to 24 h. Incubation of ^99m^Tc-complexes at 37 °C with a significant excess of the amino acids cysteine or histidine that display high affinity for the *fac*-[^99m^Tc(CO)_3_]^+^ synthon showed that more than 95% of the complexes remained intact (Figure 2).

### 2.3. Biological Evaluation

**Figure 3:**
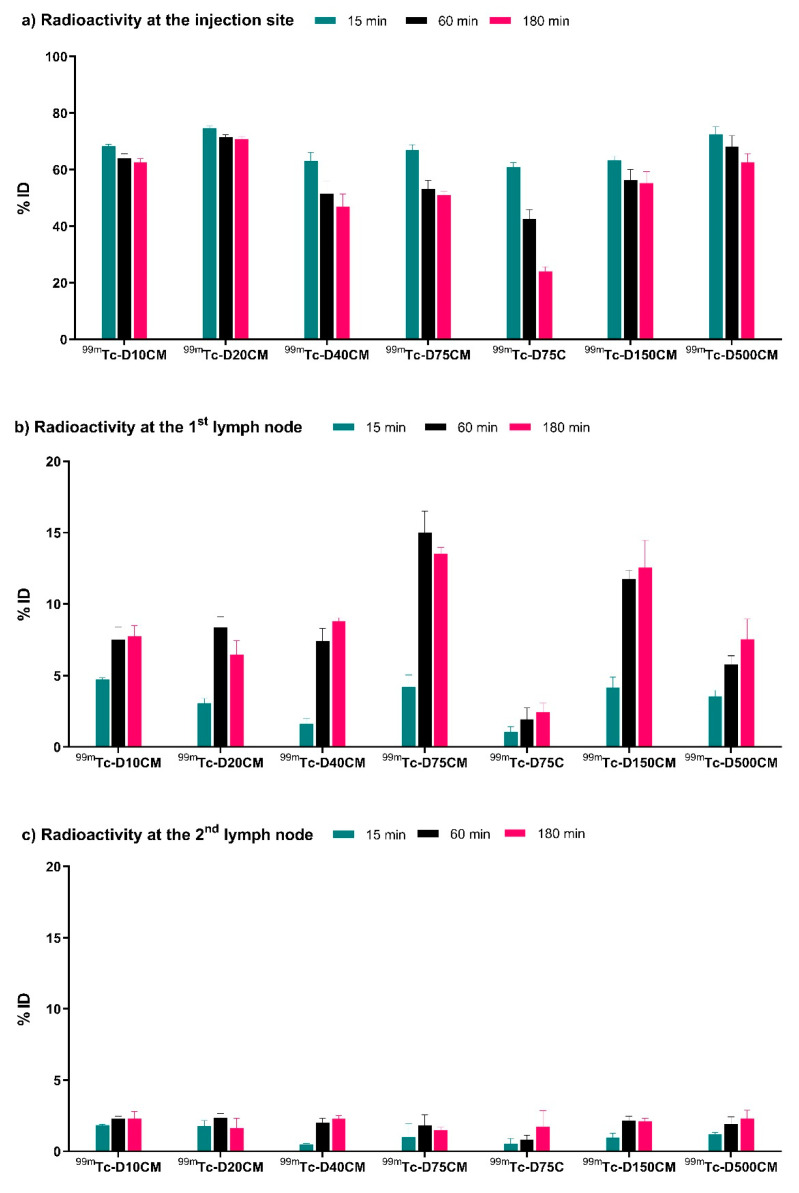
Biodistribution of radioactivity in the 1st and 2nd lymph nodes and at the injection site after subcutaneous injection of ^99m^Tc-complexes to the rear footpad of healthy Swiss Albino mice at (**a**) 15, (**b**) 60, and (**c**) 180 min.

The in vivo biodistribution of all six ^99m^Tc-labeled mannosylated dextran derivatives—^99m^Tc-D10CM, ^99m^Tc-D20CM, ^99m^Tc-D40CM, ^99m^Tc-D75CM, ^99m^Tc-D150CM, and ^99m^Tc-D500CM—was studied in healthy male Swiss Albino mice. In addition, for comparison purposes, the non-mannosylated ^99m^Tc-D75C was also tested. Each mouse received a dose of 20 μL (0.074 MBq, 0.05 μg DCM), and the biodistribution pattern of the six ^99m^Tc-labeled dextrans was studied at 15, 60, and 180 min after a subcutaneous injection via the rear footpad. The results of in vivo biodistribution studies of the ^99m^Tc-labeled mannosylated dextrans (^99m^Tc-D10CM–^99m^Tc-D500CM) are shown in Figure 3, Table 2, and Table S1.

At 15 min post injection, 25% to 37% of the injected dose (ID) cleared from the injection site (footpad). At the same time, a significant percentage of the injected activity (1.61% to 4.71% ID) localized in the popliteal lymph node, the sentinel lymph node in this study. The highest popliteal lymph node uptake was measured for the ^99m^Tc-D10CM and ^99m^Tc-D75CM derivatives, 4.71 ± 0.13% and 4.20 ± 0.84% ID, respectively, while the lowest was measured for the ^99m^Tc-D40CM derivative (1.61 ± 0.39% ID).

Slower injection site clearance was observed from 15 to 60 min post injection. In general, after 60 min, injection site uptake remained quite stable in a range from 46% to 70% ID. On the contrary, a significant increase in the popliteal lymph node uptake at 60 min p.i. was observed, ranging between 5.80% and 15.00% ID, that remained almost constant up to 180 min p.i. (6.44 to 13.53% ID). The highest values, 15.0 ± 1.5% and 13.53 ± 0.45% ID, were measured for compound ^99m^Tc-D75CM at 60 and 180 min p.i., respectively (Table 2).

The uptake of complexes in the second lymph node was in the range 0.49–1.84% ID at 15 min p.i., slowly increasing to 1.81–2.37% ID at 60 min p.i. and then remaining practically stable up to 180 min p.i. (1.49–2.30% ID). The ratio between the first and second lymph nodes was high at all time points and above 3 for most complexes. The highest ratios (8.3 and 9.0) were found for complex ^99m^Tc-D75CM at 60 and 180 min p.i., respectively.

The percentage of the radioactivity in the blood at 15 min p.i. was low (0.52–2.06% ID/g) and dropped further at 180 min p.i. (0.08–1.17% ID/g). A low concentration of radioactivity was measured in most of the organs. Specifically, the radiation in the stomach and spleen at all time points was minimal, indicating the in vivo stability of the complexes with respect to reoxidation to ^99m^TcO_4_^−^ and ^99m^TcO_2_. A significant amount of radioactivity was measured only in the liver, ranging from 1.08% to 3.72% ID/g at 15 min p.i. to 1.79% to 4.14% ID/g at 180 min p.i. The uptake in intestines was below 1.0% ID/g for all complexes at all times studied except for complex ^99m^Tc-D40CM (1.68% ID/g at 180 min p.i.), indicating minimal hepatobiliary excretion. Urinary excretion was also low, as indicated by the small percentage of radioactivity in urine (1.32–5.58% ID at 180 min p.i.).

The biodistribution of the non-mannosylated compound ^99m^Tc-D75C was also studied. The results, presented in Table 2, show superior injection site clearance with only 24.1% ID remaining in the injection site at 180 min p.i. However, only marginal uptake was observed in the popliteal lymph node (1.04–2.43% ID) and in the second lymph node (0.53–1.72% ID). In addition, high radioactivity levels were measured in the blood at all times studied (6.16–10.13% ID/g), accompanied by significant urinary excretion (22.46 ± 3.55% ID) at 180 min p.i.

**Figure 4:**
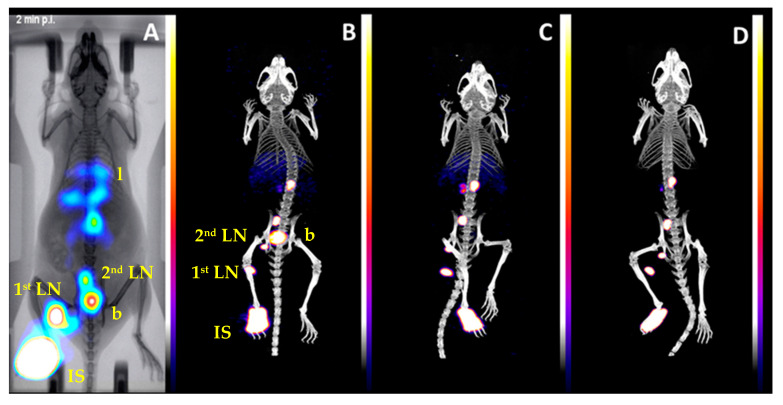
2D and 3D imaging after footpad injection of the ^99m^Tc-labeled mannosylated dextran mannose receptors targeting agent ^99m^Tc-D75CM in healthy Swiss Albino mouse. (**A**) Dynamic imaging for 30 min just after injection with γ-eye^ΤΜ^. (**B**) Tomographic imaging for 30 min at 1 h p.i., (**C**) tomographic imaging for 30 min at 3 h p.i., and (**D**) tomographic imaging for 50 min at 24 h p.i. with x-CUBE^TM^/y-CUBE^TM^, respectively. The color bar indicates the accumulation level (i.e., white being the highest and purple the lowest). 1st lymph node (1st LN); 2nd lymph node (2nd LN); Bladder (b); Liver (l); Injection site (IS).

**Figure 5:**
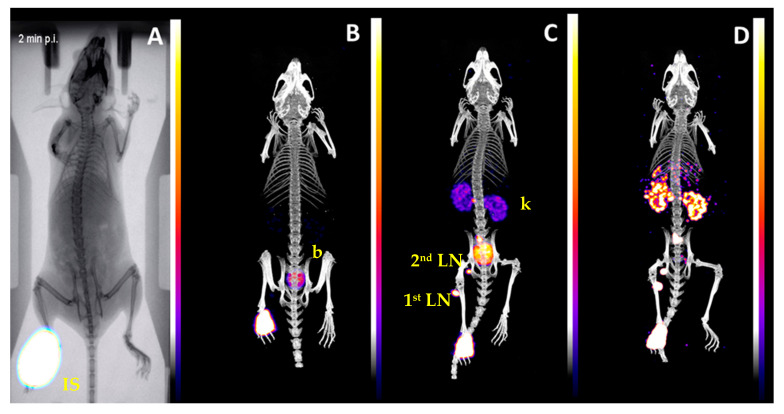
2D and 3D imaging after footpad injection of the ^99m^Tc-labeled non-mannosylated dextran agent ^99m^Tc-D75C in healthy Swiss Albino mouse. (**A**) Dynamic imaging for 30 min just after injection with γ-eye^ΤΜ^. (**B**) Tomographic imaging for 30 min at 1 h p.i., (**C**) tomographic imaging for 30 min at 3 h p.i., and (**D**) tomographic imaging for 50 min at 24 h p.i. with x-CUBE^TM^/y-CUBE^TM^, respectively. The color bar indicates the accumulation level (i.e., white being the highest and purple the lowest). 1st lymph node (1st LN); 2nd lymph node (2nd LN); Bladder (b); Kidney (k); Injection site (IS).

**Figure 6:**
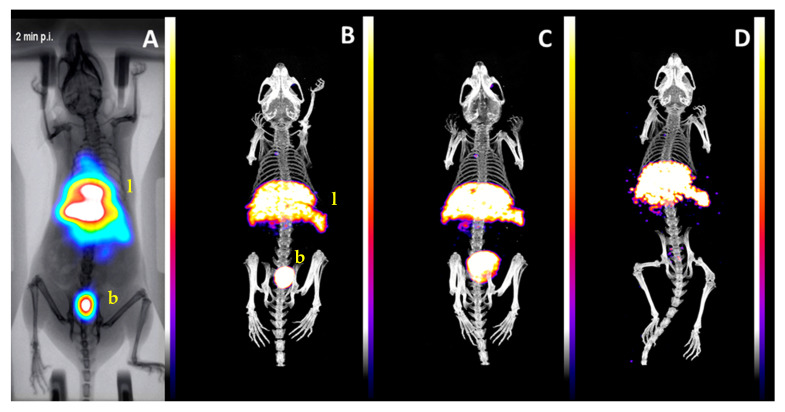
2D and 3D imaging after intravenous injection of the ^99m^Tc-labeled mannosylated dextran mannose receptors targeting agent ^99m^Tc-D75CM in healthy Swiss Albino mouse. (**A**) Dynamic imaging for 30 min just after injection with γ-eye^ΤΜ^. (**B**) Tomographic imaging for 30 min at 1 h p.i., (**C**) tomographic imaging for 30 min at 3 h p.i., and (**D**) tomographic imaging for 50 min at 24 h p.i. with x-CUBE^TM^/y-CUBE^TM^, respectively. The color bar indicates the accumulation level (i.e., white being the highest and purple the lowest). Bladder (b); Liver (l).

The in vivo imaging study of the ^99m^Tc-labeled mannosylated dextran derivative ^99m^Tc-D75CM, which showed the best overall biodistribution profile, as well as of the non-mannosylated ^99m^Tc-D75C analogue, was performed by injecting the compounds in the rear footpad. The images (Figure 4 and Figure 5) are in agreement with the biodistribution data. Minutes after injecting ^99m^Tc-D75CM, the lymph nodes were clearly delineated, and the localization persisted up to 24 h after injection. The uptake in a second and a third lymph node may be due to the saturation of the mannose receptors in the small popliteal lymph node of the mouse (1–2 mg) by the excess (2 μg, 1.8 × 10^−11^ moles) of non-radiolabeled D75CM injected in the imaging studies. On the contrary, after injecting the non-mannosylated compound ^99m^Tc-D75C, only marginal lymph node uptake was observed, followed by extensive urinary excretion. After intravenous injection of the mannosylated compound ^99m^Tc-D75CM (Figure 6), a significant percentage of radioactivity was rapidly excreted in urine, but a high and persistent localization in the liver was observed. This finding is consistent with reports in the literature, referring to the mannose receptor (CD206) as being predominantly expressed by liver endothelial cells [25].

## 3. Discussion

Sentinel lymph node detection (SLND) is rapidly entering common practice in the management of patients with tumors. SLND in nuclear medicine is performed by injecting small radiolabeled particles in the area where a tumor is located. ^99m^Tc-labeled dextrans carrying mannoses are known to be trapped in the SLN due to their recognition by the mannose receptors of lymph node macrophages. These nanocompounds consist of a 10 kDa dextran backbone, several mannose units for recognition by the mannose receptors, and a chelating agent such as DTPA in Lymphoseek, MAG_3_, or pyrazolyl-diamine for coordinating ^99m^Tc [23,24].

In previous studies [23,24], we presented the development of a novel mannosylated dextran derivative DCM20 that can be labeled with ^99m^Tc using the *fac*-[^99m^Tc(OH_2_)_3_(CO)_3_]^+^ precursor. The compound has a 10 kDa dextran as a backbone, 6 S-derivatized cysteines as efficient SNO chelators, and 24 mannose moieties for binding to mannose receptors. The biological evaluation of the ^99m^Tc-labelled ^99m^Tc(CO)_3_-DCM20 showed specific uptake in the mouse RAW 264.7 mannose receptor-bearing macrophage cells, rapid and high accumulation in the popliteal lymph node that remained almost stable up to 6 h, and fast clearance from the injection site.

In this work, we describe the synthesis, labeling with ^99m^Tc, and biological evaluation of a series of analogous mannosylated dextran derivatives (^99m^Tc-D10CM–^99m^Tc-D500CM) having a dextran backbone with a molecular weight of 10–500 kDa. It should be noted that the derivative ^99m^Tc-D10CM is essentially the same as the previously studied [23,24] compound ^99m^Tc(CO)_3_-DCM20 and is included for comparison purposes. The synthesis and characterization of the compounds D10CM–D500CM was straightforward, and no significant differences either in the percentage of allylation or in the percentage of mannosylation were noted between derivatives. Labeling with high yield and radiochemical purity was achieved with all derivatives. The stability studies of the generated ^99m^Tc-complexes showed that more than 95% of the original complexes were present after 6 h incubation with excess cysteine or histidine at 37 °C, demonstrating the strong ligation of the ^99m^Tc(CO)_3_ core to the SNO cysteine ligand and the suitability of the radiotracers for biological and imaging applications.

In vivo biodistribution studies in mice demonstrated fast injection site clearance, high uptake in the first lymph node, and relatively low uptake in the following node for all mannosylated compounds. Regarding the injection site clearance, no significant improvement was observed in comparison with the previously reported compound ^99m^Tc(CO)_3_-DCM20. However, the uptake of ^99m^Tc-D75CM in the popliteal lymph node (13–15% ID) was almost 2 times higher (Figure 3, Table S1). The specific uptake of ^99m^Tc-D75CM in the lymph node was supported by the low uptake of the non-mannosylated analogue ^99m^Tc-D75C.

In vivo imaging studies in mice with ^99m^Tc-D75CM were in agreement with the biodistribution data (Figure 4). Radioactivity cleared from the injection site and localized in the popliteal in a high percentage, allowing for precise localization and imaging. The uptake in a second and a third lymph node observed in imaging and biodistribution studies may be due to the saturation of the mannose receptors in the small popliteal lymph node of the mouse (1–2 mg) by the excess (4.5 × 10^−13^ moles) of non-radiolabeled D75CM. The latter was more noticeable in imaging studies than biodistribution because higher excess of non-radiolabeled D75CM was injected (1.8 × 10^−11^ moles). Previous studies with the^99m^Tc(CO)_3_-DCM20 in rats have shown that the leak to the second lymph node is eliminated by reducing the injected non-radiolabeled compound [24]. Thus, in larger animals or humans, the visualization of the SLN only is expected because the size of the lymph node and the number of mannose receptors will be higher. The marginal uptake in the lymph nodes after injecting the non-mannosylated compound ^99m^Tc-D75C (Figure 5) in the footpad and the accumulation of radioactivity in the liver after intravenous injection of ^99m^Tc-D75CM in mice (Figure 6) strongly support the interaction of ^99m^Tc-D75CM with mannose receptors.

Overall, the attractive biological features of ^99m^Tc-D75CM, in combination with the high radiochemical yield and stability of the compound, justify its further evaluation as a new radiopharmaceutical for sentinel lymph node detection.

## 4. Materials and Methods

All laboratory chemicals were reagent grade; they were purchased from Aldrich, Acros, or Fluka and were used without further purification. Solvents for high-performance liquid chromatography (HPLC) were HPLC-grade and degassed by a helium flux before and during use. Dextrans (D10: MW 11,800 Da, D20: MW 18,100 Da, D40: MW 40,000 Da, D75: MW 75,000 Da, D150: MW 150,000 Da, and D500: MW 500,000 Da) were purchased from Serva Electrophoresis GmbH. The helium, nitrogen, and CO gases were purchased from Air Liquide (Greece) in cylinders. The cyanomethyl 2,3,4,6-tetra-O-acetyl-1-thio-β-D-mannopyranoside was prepared according to the literature [26,27].

The ultrafiltration was performed into an ultrafiltration cell (Model 8400, Millipore Corp, Bedford, MA, USA) fitted with an ultrafiltration membrane (YM03, MW cut off 3000).

The NMR spectra were recorded in D_2_O at 25 °C on a Bruker 500 MHz Avance DRX (Bruker, Billerica, MA, USA) using sodium trimethylsilylpropanesulfonate (DSS) as an internal standard. Assignment of the spectra was based on a series of ^1^H-^1^H experiments described in detail in the literature [23].

HPLC analysis was performed on a Waters 600 chromatography system (Waters, Milford, MA, USA) coupled to a Waters 2487 Dual λ absorbance detector (Waters, Milford, MA, USA) and a Gabi gamma detector (Raytest, Germany). Separations were achieved on a Macherey-Nagel Nucleosil RP-C18 column (10 μm, 250 × 4 mm) eluted with a binary gradient system at a 1 mL/min flow rate. Mobile phase A was water containing 0.1% trifluoroacetic acid (TFA), while mobile phase B was methanol containing 0.1% TFA. The elution gradient was 0–1 min 95% A (5% B), followed by a linear gradient to 30% A (70% B) in 9 min; this composition was held for another 10 min.

Caution! Technetium-99m is radioactive, and all manipulations utilizing radioactive material were performed by authorized personnel, followed appropriate radiation safety procedures, and were conducted in supervised laboratories licensed for such work. Na^99m^TcO_4_ was obtained in physiological saline as a commercial ^99^Mo/^99m^Tc generator eluate (Ultra-Technekow™ V4 Generator, Curium Pharma, Petten, The Netherlands). The radioactive precursor *fac*-[^99m^Tc(CO)_3_(H_2_O)_3_]^+^ was prepared using a homemade kit containing 5.5 mg of NaBH_4_, 4 mg of Na_2_CO_3_, and 20 mg of Na-K tartrate, purged with CO gas before the addition of Na^99m^TcO_4_, as described in the literature [28].

### 4.1. Synthesis of Allyl-Dextran Compounds (D10A–D500A)

General method: Dextran (15.0 g), 4.0 g (1 × 10^−1^ mol) of NaOH, and 0.1 g (2.6 × 10^−4^ mol) of NaBH_4_ were dissolved in 75 mL of distilled water. The solution was warmed to 50 °C, and allyl bromide (25.4 g, 0.21 mol) was added. The pH was maintained at 11 by the addition of 2.5 N NaOH. After 4 h, the solution was neutralized (pH 7.0) with glacial acetic acid, and the allyl-dextran was purified by precipitation with ethanol. Further purification was performed by ultrafiltration. The white solid was dissolved in 50 mL water and filtered through a 5 μm filter. The filtrate was transferred into an ultrafiltration cell, and the volume was fixed to 250 mL with water and then concentrated to 15 mL by applying N_2_ gas pressure directly to the ultrafiltration cell. The retentate was diluted with 250 mL water, reconcentrated to 10 mL, and finally lyophilized. Yields ranged between 76% and 82%. All products give the same NMR peaks in D_2_O. ^1^H NMR for D10A–D500A (D_2_O, ppm), 5.99 (m, -OCH_2_C*H*=CH_2_), 5.38, 5.31 (OCH_2_CH=C*H_2_*), 5.17 (subst. dextran anomeric H-1), 4.99 (dextran anomeric H-1), 4.22 (OC*H_2_*CH=CH_2_), 4.00, 377 (dextran H-6), 3.99–3.53 (subst. dextran H-3–H-6), 3.92 (dextran H-5), 3.74 (dextran H-3), 3.60 (dextran H-2), 3.54 (dextran H-4), 3.74 (subst. dextran H-2).

### 4.2. Synthesis of Dextran-S-Cysteine Compounds (D10C–D500C)

General method: To a solution of 2.0 g of allyl dextran in 10 mL of water, 1.43 g (8.14 × 10^−3^ mol) L-cysteine hydrochloride monohydrate and 0.12 g (5.25 × 10^−4^ mol) of ammonium persulfate ((NH_4_)_2_S_2_O_8_) were added, and the resulting solution was stirred for 4 h at 50 °C under nitrogen. The pH was adjusted to 4.0 using 0.1 N NaOH, and the solution was left under stirring at room temperature for 24 h. The volume was fixed to 50 mL with 0.02 M NaOAc buffer pH 4.0, and after filtration through a 5 μm filter, the filtrate was transferred into an ultrafiltration cell. The volume was fixed to 250 mL with 0.02 M NaOAc buffer pH 4.0 and then concentrated to 10 mL by applying N_2_ gas pressure directly to the ultrafiltration cell. Subsequently, the retentate was diluted with 250 mL 0.1 M NaHCO₃ buffer, concentrated to 10 mL, as above; the retentate was diluted with 250 mL of water, reconcentrated to 10 mL, and lyophilized. Yields ranged between 70% and 75%. All products give the same NMR peaks in D_2_O. ^1^H NMR for D10C–D500C (D_2_O, ppm) 5.18 (subst. dextran anomeric H-1), 4.99 (dextran anomeric H-1), 4.00, 3.78 (dextran H-6), 3.92–3.56 (subst. dextran H-3–H-6), 3.92 (dextran H-5), 3.84, 3.78 (OC*H_2_*CH_2_CH_2_S), 3.94 (cysteine SCH_2_C*H*), 3.73 (dextran H-3), 3.59 (dextran H-2), 3.54 (dextran H-4), 3.42 (subst. dextran H-2), 3.14, 3.06 (cysteine SC*H_2_*CH), 2.72 (OCH_2_CH_2_C*H_2_*S), 1.92 (OCH_2_C*H_2_*CH_2_S).

### 4.3. Synthesis of Mannosylated Dextran-S-Cysteine Compounds (D10CM–D500CM)

General method: Τo a methanolic suspension of cyanomethyl 2,3,4,6-tetra-*O*-acetyl-1-thio-β-d-mannopyranoside (1.41 g, 3.49 × 10^−3^ mol, in 33 mL methanol), 2 mL of sodium methanoxide solution (21.6 mg, 3.99 × 10^−4^ mol) was added, and the mixture was agitated periodically. After 24 h, 15 mL of the solution was transferred to a dried recovery flask, and methanol was removed by rotary evaporation, affording 2-imino-2-methoxyethyl-1-thio-β-d-mannopyranoside as golden syrup. Immediately, a solution of DC (0.2 g) in 7.5 mL of 0.02 M sodium borate buffer pH 9.0 was added to the flask and left to react for 20 h under periodic stirring. After filtration through a 5 μm filter, the filtrate was transferred into an ultrafiltration cell. The volume was fixed to 50 mL with 0.1 M NaHCO_3_ buffer and then concentrated to 5 mL by applying N_2_ gas pressure directly to the ultrafiltration cell. Subsequently, the retentate was diluted with 50 mL deionized water, concentrated to 5 mL as above (twice), and lyophilized. Yields ranged between 80% and 85%. All products give the same NMR peaks in D_2_O, differing in relative intensities between derivatives. ^1^H NMR for D10CM–D500CM (D_2_O, ppm) 5.45, 5.41, 5.34 (mannose anomeric H-1), 5.18 (subst. dextran anomeric H-1), 4.99 (dextran anomeric H-1), 4.40, 4.33 (subst. cysteine SCH_2_C*H*), 4.10 (mannose H-2), 4.00, 3.77 (dextran H-6), 3.94–3.72 (mannose H-3–H-6) to check in TOCSY, 3.91 (dextran H-5), 4.00–3.53 (subst. dextran H-3–H-6), 3.87, 3.80 (OC*H_2_*CH_2_CH_2_S), 3.93 (free cysteine SCH_2_C*H*), 3.74 (dextran H-3), 3.58 (dextran H-2), 3.52 (dextran H-4), 3.49, 3.42 (NH=CC*H_2_*S), 3.41 (subst. dextran H-2), 3.20, 3.07, 2.94 (subst. cysteine SC*H_2_*CH), 3.14, 3.06 (free cysteine SC*H_2_*CH), 2.72 (OCH_2_CH_2_C*H_2_*S), 1.91 (OCH_2_C*H_2_*CH_2_S).

### 4.4. Synthesis of ^99m^Tc Complexes

The precursor *fac*-[^99m^Tc(CO)_3_(H_2_O)_3_]^+^ was prepared using the homemade kit, and its radiochemical purity was checked by RP-HPLC. A solution of *fac*-[^99m^Tc(CO)_3_(H_2_O)_3_]^+^ (0.5–1.0 mL, 37–740 MBq), pH 7–8, was added to a capped vial, containing 100 μg of each of the dextran compounds D10CM–D500CM or D75C. The mixture was incubated at 100 °C for 15 min and then analyzed by HPLC. For the stability and animal studies, all complexes were used without further purification.

### 4.5. In Vitro Stability Studies of ^99m^Tc Complexes

Aliquots of 400 μL (37 MBq) of ^99m^Tc complexes were added to 100 μL of histidine or cysteine (5 × 10^−2^ M) solution in PBS, pH 7.4, respectively. The samples were incubated for 1, 3, and 6 h, and aliquots were analyzed by HPLC.

### 4.6. Animal Distribution Studies

All the biodistribution studies were carried out in compliance with the Presidential Decree 56/2013 (published in the Official Government Gazette of Greece 106 A/30-4-2013) that has transposed the EU Directive 2010/63 on the protection of animals used for scientific purposes.

Groups of healthy Swiss Albino mice (male, 25 ± 3 g) under slight ether anesthesia were injected subcutaneously in the rear footpad with ^99m^Tc-labeled dextran derivatives. The preparation mixture of the ^99m^Tc-labeled compounds was diluted with saline before injection to adjust concentration. A volume of 0.02 mL (0.074 MBq, 0.05 μg DCM) was administered in three groups of three mice each to the rear footpad, while 5 min prior to sacrifice, 0.02 mL of patent blue V was also injected to facilitate lymph node visualization. After each injection, the pad was massaged for 0.5 min. At preset time intervals post-injection (15, 60, and 180 min), mice were sacrificed by cardiectomy. Lymph nodes (popliteal and inguinal) were extracted first. Other organs and samples of blood and muscle were also collected, weighed, and assayed for radioactivity. Total urine volume was collected during the experiment and added to that removed from the bladder after sacrifice. Bladder and excreted urine were not weighed. The stomach and intestines were not emptied of food contents prior to radioactivity measurements. The percentage of injected dose per organ (% ID/organ) was calculated by comparing sample radioactivity with standard solutions containing 10% of the injected dose. The calculation for blood and muscle was based on measured activity, sample weight, and body composition data (considering that blood and muscle comprise 7% and 43% of body weight). The percentage of injected dose per gram (% ID/g) was calculated by dividing the % ID/organ by the organ or tissue’s weight.

### 4.7. Imaging Studies

#### 4.7.1. Imaging Systems

Real-time, fast, dynamic screening studies were performed on a dedicated benchtop, mouse-sized, planar scintigraphy system (γ-eye^TM^ by BIOEMTECH, Athens, Greece). The system supports fusion with a digital mouse photograph. In addition, to further enhance anatomical mouse mapping to the corresponding functional information, a deep neural network was used to translate the photographic image to an artificially produced X-ray scan for anatomical co-registration [29]. The detector is based on position-sensitive photomultiplier tubes (PSPMTs), coupled to a CsI(Na) pixelated scintillator and a medium-energy lead collimator with parallel hexagonal holes, supporting a wide range of SPECT isotopes. The system’s field of view is 5 × 10 cm^2^, with a spatial resolution of ~2 mm.

For the planar imaging, healthy Swiss Albino mice were kept under isoflurane anesthesia and constant temperature of 37 °C. Short static scans were possible at different time points to provide longitudinal information on the distribution on the same animal after short anesthesia times, e.g., 10 min or less [30].

Tomographic SPECT/CT imaging was performed with y-CUBE^TM^ and x-CUBE^TM^ (Molecubes, Belgium). The y-CUBE^TM^ (Molecubes, Belgium) system provides SPECT images with a spatial resolution of 0.6 mm for mouse imaging, and the accompanying x-CUBE^TM^ (Molecubes, Belgium) can provide CT images with 50 µm resolution, operating between 35 and 80 kVp, 10 and 500 µA tube current.

Mouse imaging on the scintigraphy system was performed by keeping the mice anesthetized under isoflurane and constant temperature of 37 °C. Dynamic scans were acquired with a 2 min frame with a total of 30 min duration, based on the injected activity and system specifications.

Mouse imaging on tomographic systems was performed by keeping the mice anesthetized under isoflurane and constant temperature of 37 °C. SPECT scans were acquired with a 30–50 min duration, based on the injected activity and system specifications.

A CT scan followed each SPECT scan for co-registration purposes. The SPECT data for the Molecubes system were reconstructed through an MLEM algorithm, with 250 um voxel size and 500 iterations. CT data were reconstructed through an ISRA algorithm, with 100 µm voxel size.

#### 4.7.2. Animal Imaging Studies

For SPECT isotopes, studies with ^99m^Tc-complexes were analyzed. In one study, intravenous bolus injections of ^99m^Tc-D75CM (20 μL, ~10 MBq, ~2 μg D75CM) were performed. In all the other studies, subcutaneous injection in the footpad of lymph node targeting agent ^99m^Tc-D75CM or the non-mannosylated compound ^99m^Tc-D75C (20 μL, ~10 MBq, 2 μg D75C or D75CM) was performed. Mice were kept anesthetized during administration and imaging with isoflurane anesthesia (induction with 3–5% isoflurane flow rate and maintenance with 1–3% flow rate). The animals were imaged alive, and the studies started with 2D imaging, and the 3D scans followed right after.

For the live dynamic imaging performed with γ-eye^ΤΜ^, post-processing and quantification were performed through embedded analysis software, visual|*eyes* (BIOEMTECH, Athens, Greece).

For the tomographic images acquired with the tomographic imaging systems, post-processing was performed through third-party analysis software, VivoQuant v1.23 (Invicro LLC, Boston, MA, USA).

## 5. Conclusions

With the aim of developing the ideal radiopharmaceutical for SLND, we worked on the synthesis, labeling, and biological evaluation of a series of mannosylated dextran derivatives. Synthesis of all derivatives was successfully achieved by a multistep procedure. Labeling with ^99m^Tc using the *fac*-[^99m^Tc(CO)_3_(H_2_O)_3_]^+^ synthon resulted in each case in a single product with high radiochemical purity, and the labeled products were stable.

Biodistribution studies show that all compounds have similar or better characteristics to our previously reported compound ^99m^Tc(CO)_3_-DCM20. In addition, compound ^99m^Tc-D75CM is superior to ^99m^Tc(CO)_3_-DCM20, as the lymph node uptake is almost 2 times higher, making it a candidate for further evaluation in larger animals and clinical trials as a new radiopharmaceutical for sentinel lymph node detection.

## Figures and Tables

**Table 1:** Group density and molecular weight of D10CM–D500CM dextran derivatives.

DCM Derivative	Cysteine Moieties	Mannose Moieties	MW_calculated_
D10CM	7	19	21,297
D20CM	17	23	31,097
D40CM	29	33	59,459
D75CM	23	74	111,238
D150CM	95	160	236,320
D500CM	142	645	805,567

**Table 2:** Biodistribution of radioactivity after subcutaneous injection to the rear footpad of **^99m^Tc-D75C** and **^99m^Tc-D75CM** in mice at (a) 15, (b) 60, and (c) 180 min.

Organ	^99m^Tc-D75C			^99m^Tc-D75CM		
	15 min	60 min	180 min	15 min	60 min	180 min
Blood	6.16 ± 1.23	10.10 ± 2.19	10.13 ± 1.36	1.93 ± 0.91	0.40 ± 0.19	0.18 ± 0.04
Liver	2.11 ± 0.23	3.22 ± 0.39	3.22 ± 0.61	3.62 ± 0.87	3.70 ± 1.44	2.22 ± 0.16
Heart	0.99 ± 0.24	1.65 ± 0.56	1.88 ± 0.46	0.44 ± 0.12	0.34 ± 0.11	0.35 ± 0.03
Kidneys	5.28 ± 1.00	5.61 ± 1.16	4.92 ± 0.86	2.22 ± 0.47	0.90 ± 0.09	0.76 ± 0.13
Stomach	0.46 ± 0.10	1.32 ± 0.92	1.38 ± 0.35	0.33 ± 0.17	0.29 ± 0.04	1.08 ± 0.66
Intestines	0.40 ± 0.08	0.54 ± 0.11	1.00 ± 0.13	0.36 ± 0.12	0.33 ± 0.08	0.81 ± 0.60
Spleen	0.58 ± 0.07	1.08 ± 0.29	1.99 ± 0.47	0.73 ± 0.22	1.43 ± 1.33	1.19 ± 0.33
Muscle	0.19 ± 0.01	0.27 ± 0.01	0.34 ± 0.07	0.17 ± 0.11	0.15 ± 0.03	0.34 ± 0.11
Lungs	1.44 ± 0.07	2.32 ± 0.65	2.43 ± 0.07	0.96 ± 0.50	0.31 ± 0.06	0.38 ± 0.06
Urine *	2.47 ± 1.32	6.54 ± 4.84	22.46 ± 3.55	2.06 ± 0.74	1.69 ± 2.39	4.45 ±3.23
1st node *	1.04 ± 0.37	1.94 ± 0.80	2.43 ± 0.64	4.20 ± 0.84	15.00 ± 1.50	13.53 ± 0.45
2nd node *	0.53 ± 0.38	0.80 ± 0.34	1.72 ± 1.13	0.99 ± 0.75	1.81 ± 0.77	1.49 ± 0.20
Injection site *	60.91 ± 1.52	42.63 ± 3.27	24.05 ± 1.61	66.84 ± 1.96	53.20 ± 2.96	51.04 ± 1.20

The results are expressed as % ID/g. Mean values are reported ± SD; *n* = 3. * Mean % ID values are reported ± SD; *n* = 3.

## Data Availability

All data are available within the article or Supplementary Materials.

## Supplementary Materials

The following are available online, Figure S1: ^1^H NMR spectra (range δ_H_ 6.2−1.7) of the allyl derivative D75A, the S-derivatized cysteinyl dextran D75C, and the mannosylated dextran D75CM in D2O at 25 °C, Figure S2: ^1^H NMR spectra (range δ_H_ 5.78–1.56 ppm) of the D10CM, D20CM, D40CM, D150CM, and D500CM derivatives in D2O at 25 °C, Table S1: Biodistribution of radioactivity after subcutaneous injection to the rear footpad of ^99m^Tc-D10CM–D500CM in mice at (a) 15, (b) 60, and (c) 180 min.

## Sample Availability

Samples of the compounds are not available from the authors.
