# Supplementary material for: Comparative Study of a Series of 99mTc(CO)3 Mannosylated Dextran Derivatives for Sentinel Lymph Node Detection
Source: Molecules. 2021 Aug 7;26(16):4797. doi: 10.3390/molecules26164797 (PMC8400719; doi:10.3390/molecules26164797)
Supplement: Supplementary file 1 [file molecules-26-04797-s001.zip › molecules-1318803-supplementary.pdf]

Supporting Information

Comparative study of a series of $^{99m}Tc(CO)_3$ Mannosylated Dextran Derivatives for Sentinel Lymph Node Detection

Afroditi Papasavva [1], Antonio Shegani [1], Christos Kiritsis [1], Ioanna Roupa [1], Myrto Ischyropoulou [1], Konstantina Makrypidi [1], Irineos Pilatis [2], George Loudos [2], Maria Pelecanou [3], Minas Papadopoulos [1] and Ioannis Pirmettis [1, *]

[1]Institute of Nuclear and Radiological Sciences and Technology, Energy & Safety, NCSR '"Demokritos", 15310 Athens, Greece;

[2]BIOEMTECH Laboratories, Lefkippos Attica Technology Park - NCSR '"Demokritos", 15310 Athens, Greece;

[3]Institute of Biosciences & Applications, NCSR "Demokritos", 15310 Athens, Greece;

[*]Correspondence: ipirme@rrp.demokritos.gr; Tel.: +30 210 650 3921

Corresponding Author. Dr. Ioannis Pirmettis, Ph.D., INRASTES, NCSR "Demokritos," Ag. Paraskevi Attikis, GR-15310 Athens, Greece; Phone: +30 210 650 3921; E-mail: ipirme@rrp.demokritos.gr.

Contents

Figure S1. ^{1}H NMR spectra (range δ_H 6.2−1.7) of the allyl derivative D75A, the S-derivatized cysteinyl dextran D75C and the mannosylated dextran D75CM in D_2O at 25 °C. 3

Figure S2. ^{1}H NMR spectra (range 5.78 -1.56 ppm) of the D10CM, D20CM, D40CM, D150CM and D500CM derivatives in D_2O at 25 °C. 4

Table S1. Biodistribution of radioactivity after subcutaneous injection to the rear footpad of **^{99m}Tc-D10CM - D500CM** in mice at (a) 15, (b) 60, and (c) 180 min. 5

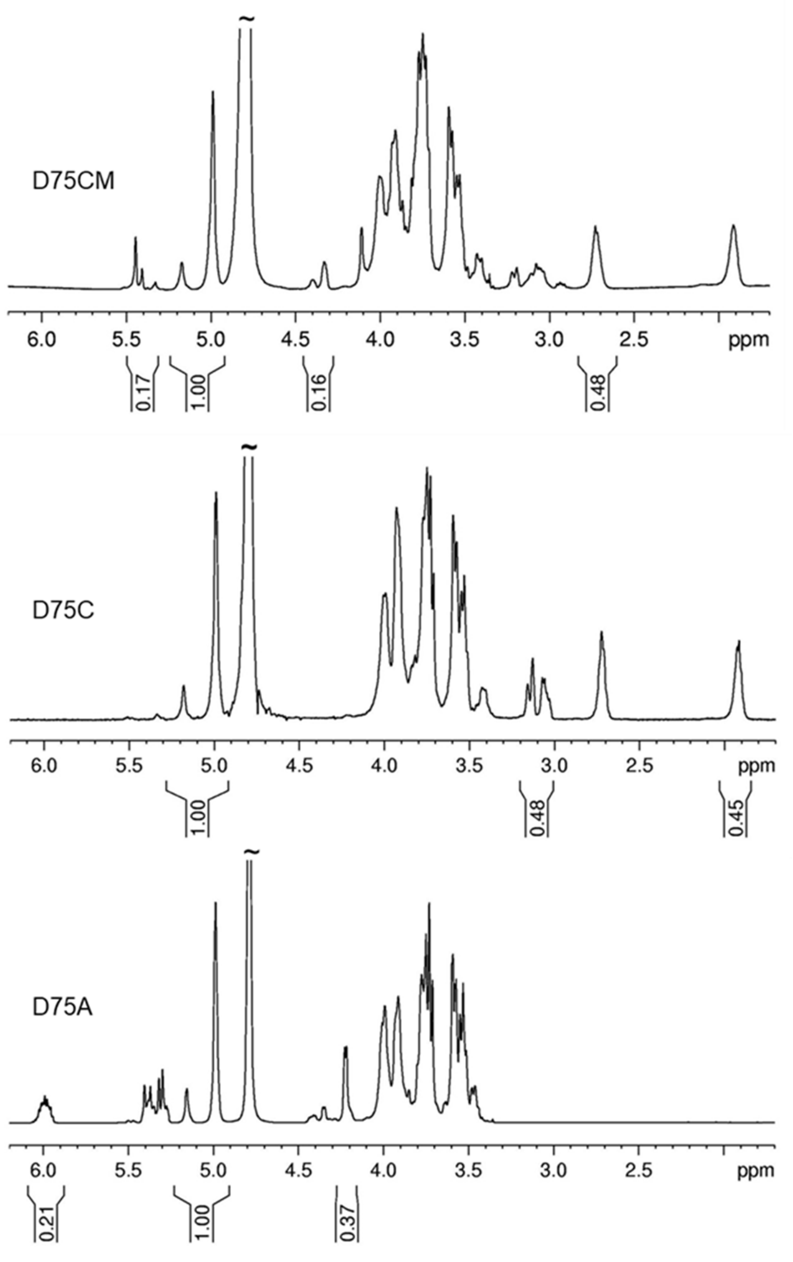

Figure S1. ^{1}H NMR spectra (range δ_H 6.2−1.7) of the allyl derivative D75A, the S-derivatized cysteinyl dextran D75C and the mannosylated dextran D75CM in D_2O at 25 °C.

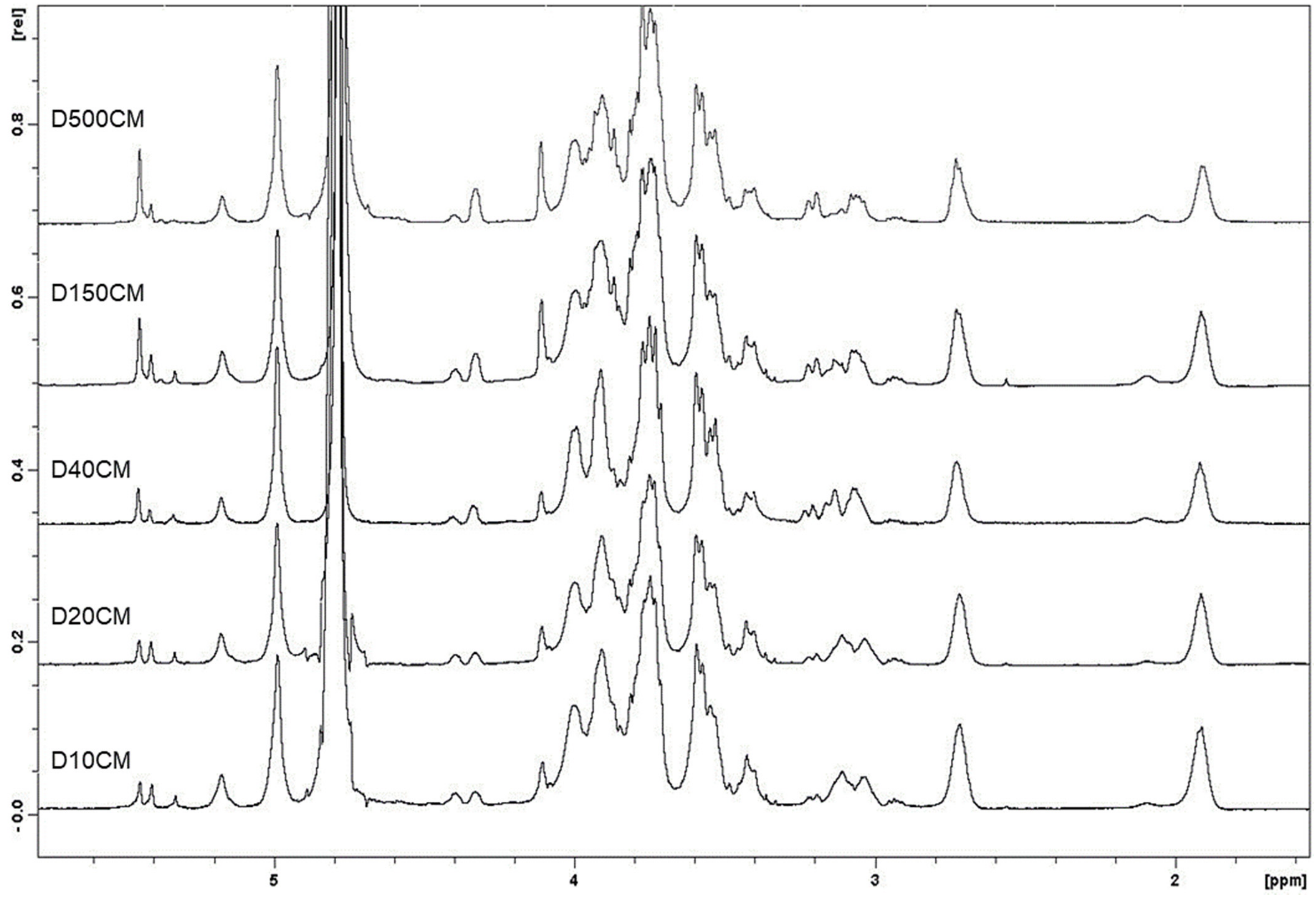

Figure S2. ^{1}H NMR spectra (range 5.78 -1.56 ppm) of the D10CM, D20CM, D40CM, D150CM and D500CM derivatives in D_2O at 25 °C.

Table S1. Biodistribution of radioactivity after subcutaneous injection to the rear footpad of **^{99m}Tc-D10CM - D500CM** in mice at (a) 15, (b) 60 and (c) 180 min.

a) 15 min p.i.	^{99m}Tc-D10CM	^{99m}Tc-D20CM	^{99m}Tc-D40CM	^{99m}Tc-D75CM	^{99m}Tc-D150CM	^{99m}Tc-D500CM
**Blood**	0.76 ± 0.18	0.52 ± 0.12	1.60 ± 0.82	1.93 ± 0.91	2.06 ± 0.14	1.53 ± 0.44
**Liver**	2.77 ± 0.42	1.08 ± 0.40	2.65 ±1.29	3.62 ± 0.87	3.72 ± 0.75	1.37 ± 0.27
**Heart**	0.87 ± 0.24	0.18 ± 0.02	0.72 ±0.21	0.44 ± 0.12	0.57 ± 0.07	0.29 ± 0.09
**Kidneys**	3.12 ± 0.38	1.59 ± 0.11	2.20 ± 0.87	2.22 ± 0.47	3.06 ± 0.86	1.52 ± 0.51
**Stomach**	0.15 ± 0.02	0.07 ± 0.03	0.46 ±0.28	0.33 ± 0.17	0.24 ± 0.09	0.77 ± 0.30
**Intestines**	0.22 ± 0.02	0.06 ± 0.01	0.33 ±0.11	0.36 ± 0.12	0.34 ± 0.08	0.21 ± 0.11
**Spleen**	0.52 ± 0.01	0.31 ± 0.08	0.81 ±0.48	0.73 ± 0.22	0.38 ± 0.18	0.50 ± 0.23
**Muscle**	0.11 ± 0.00	0.06 ± 0.02	0.29 ±0.18	0.17 ± 0.11	0.18 ± 0.04	0.11 ± 0.03
**Lungs**	0.32 ±0.08	0.19 ± 0.12	1.33 ± 0.59	0.96 ± 0.50	1.11 ± 0.40	0.62 ± 0.21
**Urine***	1.94 ± 0.30	0.95 ± 0.37	0.76 ± 0.27	2.06 ± 0.74	2.03 ± 0.52	1.64 ± 0.18
**1st node***	4.71 ± 0.13	3.04 ± 0.36	1.61 ± 0.39	4.20 ± 0.84	4.16 ± 0.75	3.53 ± 0.44
**2nd node***	1.84 ± 0.05	1.77 ± 0.39	0.49 ± 0.07	0.99 ± 0.75	0.97 ± 0.30	1.22 ± 0.10
**Injection site***	68.24 ± 0.70	74.68 ± 0.72	63.11 ± 2.94	66.84 ± 1.96	63.28 ± 1.51	72.47 ± 2.72

b) 60 min p.i.	^{99m}Tc-D10CM	^{99m}Tc-D20CM	^{99m}Tc-D40CM	^{99m}Tc-D75CM	^{99m}Tc-D150CM	^{99m}Tc-D500CM
**Blood**	0.31 ± 0.02	0.10 ± 0.03	1.62 ± 0.23	0.40 ± 0.19	0.92 ± 0.03	0.75 ± 0.15
**Liver**	3.86 ± 0.55	1.38 ± 0.13	1.70 ± 0.71	3.70 ± 1.44	3.76 ± 0.22	2.72 ± 0.25
**Heart**	0.27 ± 0.03	0.14 ± 0.06	0.56 ± 0.10	0.34 ± 0.11	0.28 ± 0.04	0.18 ± 0.02
**Kidneys**	1.84 ± 1.15	0.40 ± 0.03	1.02 ± 0.51	0.90 ± 0.09	1.97 ± 0.47	0.92 ±0.15
**Stomach**	0.32 ± 0.11	0.05 ± 0.04	0.85 ± 0.33	0.29 ± 0.04	0.14 ± 0.08	0.86 ± 0.54
**Intestines**	0.31 ± 0.03	0.11 ± 0.01	0.62 ± 0.08	0.33 ± 0.08	0.73 ± 0.31	0.28 ± 0.03
**Spleen**	0.95 ± 0.29	0.44 ± 0.14	1.92 ± 0.40	1.43 ± 1.33	1.82 ± 0.39	0.78 ± 0.12
**Muscle**	0.10 ± 0.02	0.06 ± 0.02	0.19 ± 0.03	0.15 ± 0.03	0.09 ± 0.03	0.06 ± 0.01
**Lungs**	0.24 ± 0.01	0.09 ± 0.01	0.91 ± 0.05	0.31 ± 0.06	0.39 ±0.12	0.29 ± 0.03
**Urine***	0.79 ± 0.70	1.73 ± 1.22	0.85 ± 1.21	1.69 ± 2.39	1.48 ± 0.42	2.31 ± 1.55
**1st node***	7.49 ± 0.88	8.36 ± 0.73	7.40 ± 0.89	15.00 ± 1.50	11.73 ± 0.64	5.80 ± 0.58
**2nd node***	2.28 ± 0.19	2.37 ± 0.30	2.00 ± 0.31	1.81 ± 0.77	2.17 ± 0.29	1.93 ± 0.49
**Injection site***	64.03 ± 1.56	71.41 ± 0.92	51.59 ± 4.25	53.20 ± 2.96	56.34 ± 3.69	68.15 ± 3.88

c) 180 min p.i.	^{99m}Tc-D10CM	^{99m}Tc-D20CM	^{99m}Tc-D40CM	^{99m}Tc-D75CM	^{99m}Tc-D150CM	^{99m}Tc-D500CM
**Blood**	0.23 ± 0.01	0.08 ± 0.02	1.17 ± 0.02	0.18 ± 0.04	0.29 ± 0.12	0.20 ± 0.02
**Liver**	2.75 ± 0.59	1.79 ± 0.35	3.85 ± 2.03	2.22 ± 0.16	4.14 ± 1.94	3.30 ± 0.34
**Heart**	0.19 ± 0.00	0.09 ± 0.03	0.40 ± 0.05	0.35 ± 0.03	0.27 ± 0.06	0.10 ± 0.01
**Kidneys**	1.17 ± 0.66	0.51 ± 0.21	0.76 ± 0.92	0.76 ± 0.13	1.74 ± 0.39	0.52 ± 0.09
**Stomach**	0.31 ± 0.10	0.25 ± 0.17	1.10 ± 0.44	1.08 ± 0.66	0.48 ± 0.37	1.02 ± 0.29
**Intestines**	0.27 ± 0.02	0.29 ± 0.18	1.68 ± 0.13	0.81 ± 0.60	0.63 ± 0.13	0.32 ± 0.04
**Spleen**	0.39 ± 0.08	0.39 ± 0.10	1.13 ± 0.51	1.19 ± 0.33	1.82 ± 0.97	1.68 ± 0.94
**Muscle**	0.08 ± 0.02	0.03 ± 0.01	0.75 ± 0.41	0.34 ± 0.11	0.07 ± 0.01	0.02 ± 0.01
**Lungs**	0.18 ± 0.02	0.09 ± 0.03	0.30 ± 0.27	0.38 ± 0.06	0.26 ± 0.05	0.13 ± 0.01
**Urine***	4.37 ± 2.19	1.32 ± 1.83	1.80 ± 1.27	4.45 ±3.23	5.58 ± 1.72	5.15 ± 0.60
**1st node***	7.73 ± 0.75	6.44 ± 1.01	8.79 ± 0.25	13.53 ± 0.45	12.54 ± 1.93	7.53 ± 1.41
**2nd node***	2.29 ± 0.52	1.65 ± 0.69	2.28 ± 0.24	1.49 ± 0.20	2.11 ± 0.23	2.30 ± 0.59
**Injection site***	62.60 ± 1.23	70.78 ± 0.97	46.84 ± 4.57	51.04 ± 1.20	55.20 ± 4.12	62.52 ± 3.19

The results are expressed as % ID/g. Mean values are reported ± SD; n = 3. *Mean % ID values are reported ± SD; n = 3.
